# Tolerance and dependence to Δ^9^-tetrahydrocannabinol in rhesus monkeys: Activity assessments

**DOI:** 10.1371/journal.pone.0209947

**Published:** 2019-03-12

**Authors:** Jenny L. Wilkerson, David R. Schulze, Lance R. McMahon

**Affiliations:** 1 Department of Pharmacodynamics, The University of Florida, College of Pharmacy, Gainesville, Florida, United States of America; 2 Department of Pharmacology, The University of Texas Health Science Center at San Antonio, San Antonio, Texas, United States of America; Scripps Research Institute, UNITED STATES

## Abstract

*Cannabis* withdrawal upon discontinuation of long-term, heavy *Cannabis* use is reported in humans; however, methods to establish the nature and intensity of cannabinoid withdrawal, especially directly observable signs, have not been widely established. This study quantified activity in the home cage of rhesus monkeys and examined the extent to which activity can be used to quantify tolerance to and dependence on Δ^9^-tetrahydrocannabinol (Δ^9^-THC). Home-cage activity was measured in one group that received Δ^9^-THC (1 mg/kg s.c.) every 12 h (i.e., chronic Δ^9^-THC), and a second group that received Δ^9^-THC (0.1 mg/kg i.v.) once every 3 days (i.e., intermittent Δ^9^-THC). Treatment was temporarily discontinued in the chronic Δ^9^-THC group and the effects of rimonabant and Δ^9^-THC were examined in both groups. Activity counts were highest during the day (lights on 0600–2000 h) and were lower at night. Rimonabant (0.1–3.2 mg/kg i.v.) dose-dependently increased activity (maximum 20-fold) in the chronic Δ^9^-THC group but did not significantly alter activity in the intermittent Δ^9^-THC group. Δ^9^-THC (0.32–3.2 mg/kg i.v.) dose-dependently decreased activity counts (maximum 4-fold) in both groups but was somewhat more potent in the intermittent as compared with the Δ^9^-THC group. Discontinuation of Δ^9^-THC treatment resulted in an immediate (i.e., within 24 h) and time-related increase in activity. The time-related increase in home-cage activity upon abrupt discontinuation of chronic Δ^9^-THC treatment, as well as the effects of rimonabant to increase activity in monkeys receiving chronic, but not intermittent, Δ^9^-THC treatment, are consistent with signs of physical dependence on Δ^9^-THC in primates.

## Introduction

As evidenced by a withdrawal syndrome after abrupt discontinuation of use, long-term cannabis use results in dependence (see [1,2] for reviews). Cannabis withdrawal consists primarily of self-reported symptoms including anger, anxiety, depressed mood, irritability, headaches, tension, stomach pain, and strange dreams. Directly observable signs of cannabis withdrawal are typically less prevalent than self-reported symptoms. These observable signs include aggression, sleep difficulty, decreased food intake, weight loss, and sweating. Cannabis withdrawal signs and symptoms typically emerge within 24 h after abrupt discontinuation of cannabis use and subside within 1–2 weeks. As individuals use cannabis to avoid or alleviate withdrawal, cannabis withdrawal, although not life-threating, is clinically significant [3].

Nonhuman primates have been used in studies of dependence and withdrawal resulting from a variety of drugs including Δ^9^-tetrahydrocannabinol (Δ^9^-THC), the drug primarily responsible for the behavioral effects of cannabis. However, Δ^9^-THC dependence has not been unanimously reported, as observable withdrawal signs in rhesus monkeys were not readily evident after discontinuation of Δ^9^-THC treatment (2 mg/kg/day for 30 days; [4]). Further, cardiovascular function in rhesus monkeys did not show evidence of a withdrawal syndrome after discontinuation of Δ^9^-THC treatment (2 mg/kg/day for 3 weeks; [5]). Observable signs of withdrawal also did not emerge upon termination of daily treatment with another cannabinoid agonist (levonantradol) in rhesus monkeys [6]. Meanwhile, in contrast to these negative findings, abrupt discontinuation of Δ^9^-THC (2 mg/kg/day for 3 weeks) increased some observable behaviors above baseline [7], and there was a time-related disruption in operant responding for food when continuous i.v. administration of Δ^9^-THC (0.05 mg/kg/h) was discontinued [8]. Collectively, these studies not only illustrate the mixed results typical of pre-clinical studies examining Δ^9^-THC dependence, but they also underscore the relatively mild nature of cannabis withdrawal as compared to withdrawal from other drugs such as ethanol and opioids.

CB_1_ receptor-selective antagonists such as rimonabant have been useful in pre-clinical studies of cannabinoid dependence. However, the use of rimonabant has not been free of limitations. Rimonabant surmountably antagonized many of the effects of cannabinoid agonists [9]. Moreover, rimonabant alone produced behavioral effects including hyperactivity, scratching, and wet-dog shakes in rodents [9,10], as well as headshaking and increased heart rate in rhesus monkeys [11]. Although rimonabant produced many of these effects under conditions of chronic Δ^9^-THC treatment, the ability of rimonabant to induce these effects in the absence of cannabinoid treatment suggests that not all of these effects are directly related to dependence and withdrawal. Conversely, rimonabant notably produced headshakes in rodents, among other effects, during chronic Δ^9^-THC treatment, but not in the absence of Δ^9^-THC treatment [10]. Further, rimonabant paired with Δ^9^-THC administration produced a distinctive discriminative stimulus effect in rhesus monkeys [11]. Moreover, in this study, rimonabant induced headshakes in Δ^9^-THC treated animals, which was only observed upon abrupt discontinuation of Δ^9^-THC treatment [11], indicating that some effects, such as headshakes, are signs of Δ^9^-THC withdrawal.

One goal of the present study was to examine the extent to which movement resulting from the general activity (i.e., motor activity) of individually housed rhesus monkeys in the home cage is sensitive to Δ^9^-THC dependence and withdrawal. The Δ^9^-THC treatment (1 mg/kg/12 h s.c.) used here was the same as that used to establish and maintain a rimonabant discriminative stimulus [11]. A second and related goal was to compare abrupt discontinuation of Δ^9^-THC treatment to administration of rimonabant during Δ^9^-THC treatment. Motor activity was assessed during Δ^9^-THC treatment, after treatment was abruptly discontinued, and for several weeks after resumption of Δ^9^-THC treatment, as well as after various doses of rimonabant during Δ^9^-THC treatment. Previous studies from our laboratory demonstrate that, compared to intermittent non-daily exposure in rhesus monkeys, greater tolerance to the behavioral effects of Δ^9^-THC develops from daily exposure [12]. Thus, a long history (i.e., several years) of daily Δ^9^-THC treatment might affect the outcome of (i.e., increase) the withdrawal behavior observed. To account for this, a separate group of monkeys was used to control for Δ^9^-THC treatment. Monkeys in the second group had discriminated a relatively small dose (0.1 mg/kg i.v.) of Δ^9^-THC administered once every 3 days on average for years. While the second group was not a negative control, the marked difference in Δ^9^-THC treatment dose and frequency was expected to provide a sufficient control for the impact of daily Δ^9^-THC treatment on the effects of rimonabant.

## Materials and methods

### Subjects

A total of ten adult rhesus monkeys (*Macaca mulatta*; five female and five male) were used in these studies. Monkeys were housed separately on a 14-h light/10-h dark schedule (lights on at 0600 h), were maintained at 95% free-feeding weight (range 5.6–10.1 kg) with a diet consisting of fresh fruit (apples, bananas, and oranges), peanuts, and primate chow (High Protein Monkey Diet, Harlan Teklad, Madison, WI). Monkeys received food at 1600 h each day and water was available continuously in the home cage. Monkeys received non-cannabinoids and cannabinoids in previous studies [11,13]. Experiments were approved and conducted in accordance with the Institutional Animal Care and Use Committee for The University of Texas Health Science Center at San Antonio. Additionally, all subjects received environmental enrichment as determined by the Institutional Animal Care and Use Committee for The University of Texas Health Science Center at San Antonio. As experiments were conducted 7 days/week, animals were monitored daily for signs of distress, as detailed in the “Guidelines for the Care and Use of Mammals in Neuroscience and Behavioral Research” [14]. If a subject exhibited discomfort or distress, it was removed from the study and allowed to fully recover before being continued on other studies. At the end of these experiments, monkeys were returned to their colony and continued with other studies.

### Surgery

All monkeys underwent catheter surgery, where using sterile surgical techniques, a catheter (heparin coated polyurethane, od = 1.68 mm, id = 1.02 mm, Instech Solomon, Plymouth Meeting, PA) was inserted 5 cm into a femoral or subclavian vein under anesthesia with ketamine (10 mg/kg i.m.) and isoflurane (1.5–3.0% inhaled via facemask). Suture silk (coated vicryl, Ethicon Inc., Somerville, New Jersey) secured the catheter to the vessel and was used to ligate the section of the vessel adjacent to the catheter insertion. The opposite end of the catheter was attached to a vascular access port (Mida-cbas-c50, Instech Solomon), which was located s.c. in the mid-scapular region of the back. After surgery, subjects were given meloxicam and buprenorphine as anti-inflammatory and analgesic interventions for a minimum of 2 days and were monitored daily for signs of distress.

### Apparatus

Activity was monitored in stainless-steel home cages measuring 33 inches wide × 27 inches deep × 32 inches high. The activity monitor was an ActiCal Activity Monitor 64 K Memory Waterproof (Philips Respironics Mini-Mitter Company, Inc., Bend OR). The monitor was attached to an Actiwatch Animal Case (Phillips Respironics), which was fastened to the Primate Products collar worn around the neck. The Actical Data Acquisition algorithm involves an electric sensor, which generates a voltage when it undergoes a change in acceleration. Thirty-two times per second, the filtered, amplified voltage is converted to a digital value, is used to adjust a running baseline value, and is added to a one second accumulated value. Every second, the one-second sum is divided by four and added to a resultant accumulated value for the epoch. At the end of each epoch, the accumulated activity value is compressed into an 8-bit value and stored in Actical memory. The data are downloaded by Windows software, the 8-bit values are decompressed to 15-bit raw activity counts, and the Actical-specific calibration constant is applied to the raw activity counts, resulting in calibrated activity data.

### Drugs

Rimonabant and Δ^9^-THC (100 mg/ml in absolute ethanol; The Research Technology Branch of the National Institute on Drug Abuse, Rockville, MD) were dissolved in a mixture of 1 part absolute ethanol, 1 part Emulphor-620 (Rhodia Inc., Cranbury, NJ), and 18 parts physiologic saline. Of note, this vehicle is standard in monkeys [8,15]. The highest dose of ethanol thus administered, expressed as w/w was 0.01 g/kg, which is markedly smaller than the amount required to produce ethanol dependence in a monkey. For the largest dose (3.2 mg/kg) of rimonabant, the vehicle was 1 part absolute ethanol, 2 parts Emulphor-620, and 7 parts physiologic saline. Rimonabant and Δ^9^-THC were administered in a volume of 0.1 ml/kg for subcutaneous administration and 0.1–1 ml/kg for intravenous administration.

### Experimental design

Five adult rhesus monkeys (three female and two male) that had previously received 1 mg/kg/12 h of Δ^9^-THC s.c. daily, for at least two years, were available for both the discontinuation and the i.v. Δ^9^-THC/rimonabant studies. However, due to poor health, two monkeys from the daily Δ^9^-THC (1 mg/kg/12 h s.c.) group were excluded from the discontinuation study. Thus, three monkeys (one male and two female) were used for the discontinuation study. Based upon our previous rhesus monkey studies, this sample size is sufficiently powered for studies involving the behavioral effects of daily Δ^9^-THC [13].

Five additional adult rhesus monkeys (three male and two female) that had previously received 0.1 mg/kg of Δ^9^-THC i.v. on average once every 3 days were also used in the i.v. Δ^9^-THC/rimonabant studies. These monkeys were not studied in the discontinuation study, because their THC treatments were not homogenized like the chronic Δ^9^-THC monkeys whose treatment was time locked and identical for all subjects. Specifically, these monkeys received Δ^9^-THC due to involvement in other Δ^9^-THC drug discrimination behavioral experiments, as published elsewhere. Thus, the animals received Δ^9^-THC on different days according to whether they passed the training conditions for drug discrimination. Further, this intermittent dosing of 0.1 mg/kg of Δ^9^-THC i.v. does not produce tolerance to the physiological effects of higher doses of Δ^9^-THC and as previous studies do not report overt withdrawal signs, does not produce dependence [12].

### Discontinuation study

One week before discontinuation three monkeys from the daily Δ^9^-THC group were fitted with an activity monitor and data was collected to establish an activity baseline. During discontinuation of Δ^9^-THC treatment, monkeys received vehicle instead of Δ^9^-THC starting at 6:15 PM; the following day was defined as the first full day of discontinuation (Day 1). Monkeys received vehicle for the next 19 days (Days 2–20) and at 6:15 AM the following day (Day 21). Δ^9^-THC treatment was resumed at 6:15 PM and the following day was defined as the first full day of resumed Δ^9^-THC treatment. To administer 1 mg/kg of subcutaneous Δ^9^-THC or vehicle at 6:15 AM and 6:15 PM, monkeys were pulled toward the front of the home cage via a retractable back wall, a process requiring less than 1 min.

### Intravenous Δ^9^-THC and rimonabant

As described above, two groups of monkeys (n = 5 per group) were used. One group received daily Δ^9^-THC (1 mg/kg/12 h) and the other group received intermittent Δ^9^-THC (0.1 mg/kg i.v. every 3 days on average). For intravenous injections of Δ^9^-THC and rimonabant, monkeys were removed from the home cage and were seated in chairs (Model R001, Primate Products, Miami, FL) at 12:10 PM. A dose of drug or vehicle was administered and monkeys were returned to the home cage at 12:15 PM. Activity was measured until 2:15 PM.

### Treatment order

For monkeys undergoing both discontinuation and i.v. rimonabant studies, discontinuation studies were conducted prior to beginning studies with i.v. rimonabant. The treatment order was nonsystematic, i.e., was not ascending, descending, or the same for all monkeys. Doses were chosen from those shown to produce dose-dependent cardiovascular and discriminative stimulus effects [11].

### Data analyses

An activity count was generated every 15 s and these were cumulated (i.e., 1, 2, or 24 h) for data presentation and analysis. For the discontinuation study, daily activity patterns for individual monkeys were plotted by cumulating activity in 1-h bins and showing these data across a 24-h period during daily Δ^9^-THC treatment, after discontinuation, and upon its resumption. These data were first analyzed via a one-way repeated measures analysis of variance (ANOVA), with Tukey post hoc analysis following a significant ANOVA (P < 0.05). The data was also expressed as a mean of the 5 days during Δ^9^-THC treatment immediately preceding discontinuation, the first 5 days of discontinuation, the last 5 days of discontinuation, and the first 5 days of resumption of daily Δ^9^-THC treatment and analyzed via a one-way ANOVA with Holms-Sidak post hoc analysis following a significant ANOVA (P < 0.05). These data are expressed as the mean +/- standard error of the mean (SEM).

To examine the effects of intravenous Δ^9^-THC (0.1–3.2 mg/kg) and rimonabant (0.1–3.2 mg/kg), data were cumulated for 2 h post-injection and were plotted and analyzed as a percentage of the control. For each monkey, activity counts were cumulated over 2 h following administration of vehicle; data from two separate vehicle tests were averaged for further analysis. Straight lines were simultaneously fit to individual dose-response data with linear regression. The slopes and intercepts of the dose-response functions determined in the chronic and intermittent Δ^9^-THC groups were compared to determine whether a single line was sufficient to describe the data (i.e. the two dose-response functions were not significantly different) or two lines were needed to describe the data. Activity counts were averaged per individual and expressed as a percentage of control. These data were then analyzed via a one-way repeated measures analysis of variance (ANOVA), with Fisher’s post hoc analysis of each experimental treatment group compared to vehicle following a significant ANOVA (P < 0.05), and plotted as a mean ± S.E.M. for the group. The computer program GraphPad Prism version 5.0 (GraphPad Software Inc., San Diego, CA) was used in all statistical analyses.

## Results

### Daily Δ^9^-THC treatment: Control activity counts

**Fig 1:**
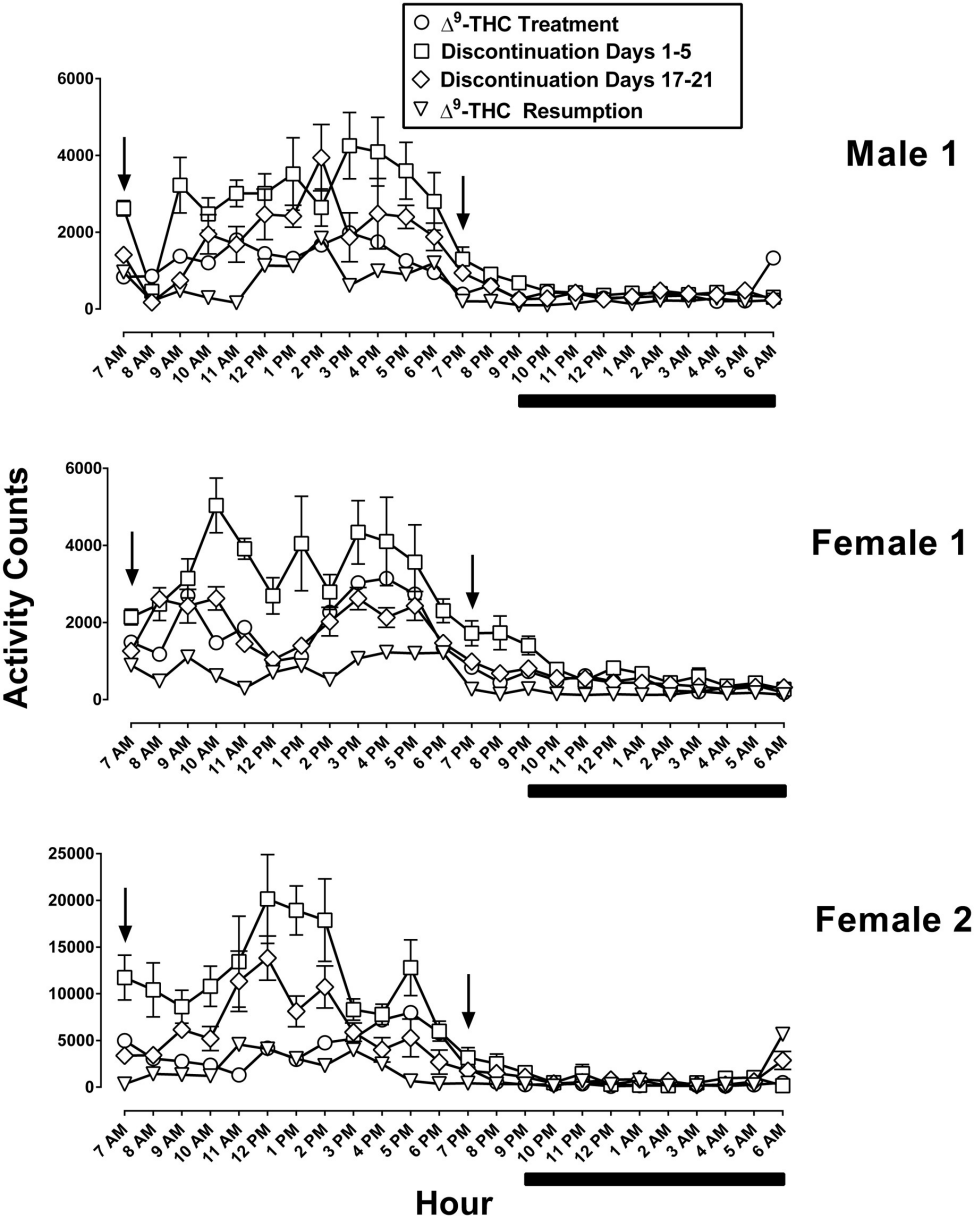
Absolute activity counts cumulated in 1-h bins and expressed for each h of the 24-h light/dark period. Each panel represents data from an individual monkey. Symbols are an average of 5 consecutive days including the 5 days immediately preceding discontinuation of Δ^9^-THC treatment (circles), the first and last 5 days of discontinued treatment (squares and diamonds, respectively), as well as the first 5 days of resumption of Δ^9^-THC treatment (triangles). The arrows indicate the time of Δ^9^-THC (1 mg/kg) or vehicle administration; vehicle was administered instead of Δ^9^-THC during the discontinuation phase. Below the abscissae the solid line denotes the dark period. Note the larger range of values on the ordinate of the lower panel as compared with the middle and top panels.

In monkeys that had received 1 mg/kg of Δ^9^-THC every 12 h for at least two years, activity counts were highest during the light period and lowest during the night (Fig 1; each panel shows data from an individual). Activity varied among monkeys, with two monkeys showing lower overall activity (Fig 1 top and middle) than a third monkey (Fig 1 bottom; note the different range on the ordinate). When expressed as cumulative activity counts per h, the highest activity occurred between 3–5 pm. The individual maximum values were 1987, 3143, and 7977 counts per h (Fig 1 top, middle, and bottom respectively, circles). In contrast, the activity counts during the night were as low as 200, 181, and 106 in each respective monkey. In general, a period of inactivity began at 7 pm (2 h before dark onset) and ended at 5 am. Cumulative 24-h activity was relatively stable during the 5 days immediately preceding discontinuation of Δ^9^-THC treatment, i.e., daily activity did not deviate more than -2% (day 3) to +12% (day 4) of the 5-day running mean. For the 95% confidence limits calculated for each day of the 5-day running mean, the maximum upper limit was 168% (day 5) and the minimum lower limit was 54% (day 2).

### Daily Δ^9^-THC treatment: Discontinuation and resumption

**Fig 2:**
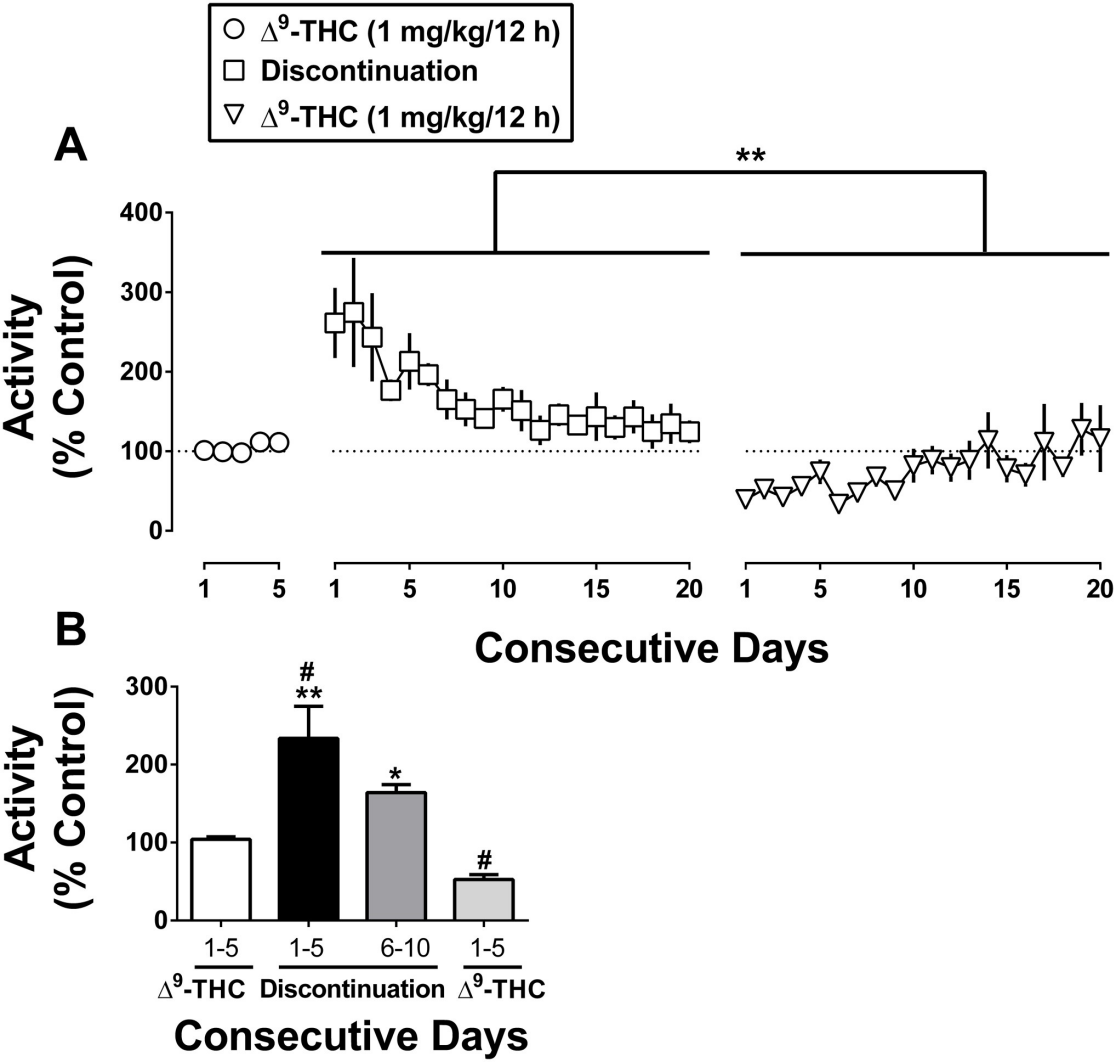
Activity counts from Fig 1 cumulated daily (i.e., for 24 h) and expressed as a percentage of control. Consecutive days at various stages including before, during, and after discontinuation of Δ^9^-THC treatment (top panel). The mean activity counts for the 5 consecutive days immediately preceding discontinuation of Δ^9^-THC treatment, day 1–5 and day 6–10 Δ^9^-THC discontinuation, and day 1–5 resumption of Δ^9^-THC are also shown (bottom panel). # P < 0.05 vs. the days preceding discontinuation of Δ^9^-THC. *P < 0.05 and **P < 0.01 vs. resumption of Δ^9^-THC. Data reflect mean ± SEM, n = 3 monkeys.

Both Figs 1 and 2 show the effects of discontinuation and resumption of Δ^9^-THC in the same monkeys expressed as individual data (Fig 1) and combined (Fig 2). Overall, discontinuation of daily Δ^9^-THC produced a significant increase in activity counts [main effect of treatment: F_2,4_ = 23.89, P < 0.05, (Fig 2 top)]. The activity levels on day 1–5 of discontinuation are significantly higher than those of the previous 5 days of daily Δ^9^-THC [P < 0.05, (Fig 2 bottom)]. Inspection of individual data in 1-h bins over the 24-h, light/dark period demonstrates that the increase in activity counts occurred predominantly during the light period (Fig 1, squares). The increase was evident at the beginning of the light period (7 AM) and throughout the day. When monkeys received Δ^9^-THC daily, they were relatively inactive by 7–8 pm (Fig 1, circles). During the first 5 days of discontinuation, however, activity remained high at 7–8 pm (Fig 1, squares). During the night, there was a trend for activity to be increased during brief intervals in two monkeys (Fig 1, squares over the black line in the middle and lowest panels) but not a third monkey (Fig 1 top).

Activity levels on days 1–5 and 6–10 of discontinuation are significantly higher than those of the first 5 days of the resumption of daily Δ^9^-THC [P < 0.001 and P < 0.05, respectively (Fig 2 bottom)]. Thus, resumption of Δ^9^-THC treatment resulted in a marked decrease in activity counts when compared to overall discontinuation [P < 0.001, (Fig 2 top, triangles)]. The decreased activity upon resumption of Δ^9^-THC treatment was most prominent during the light period (Fig 1, triangles). Additionally, mean activity levels on day 1–5 of resumption were significantly lower than those for the 5 consecutive days immediately preceding discontinuation of Δ^9^-THC [P < 0.05, (Fig 2 bottom)].

### Effects of Δ^9^-THC and rimonabant

In the five monkeys receiving chronic Δ^9^-THC (1 mg/kg/12 h), activity counts measured over a 2-h period following intravenous vehicle were 832, 3532, 3703, 6800, and 14433 for each respective monkey. In a separate group of monkeys (n = 5) receiving intermittent Δ^9^-THC (0.1 mg/kg i.v. every 3 days on average), activity counts were somewhat higher, especially in two monkeys (4910, 5928, 15531, 25651, and 56430 for each respective monkey), as compared with monkeys receiving chronic Δ^9^-THC daily.

**Fig 3:**
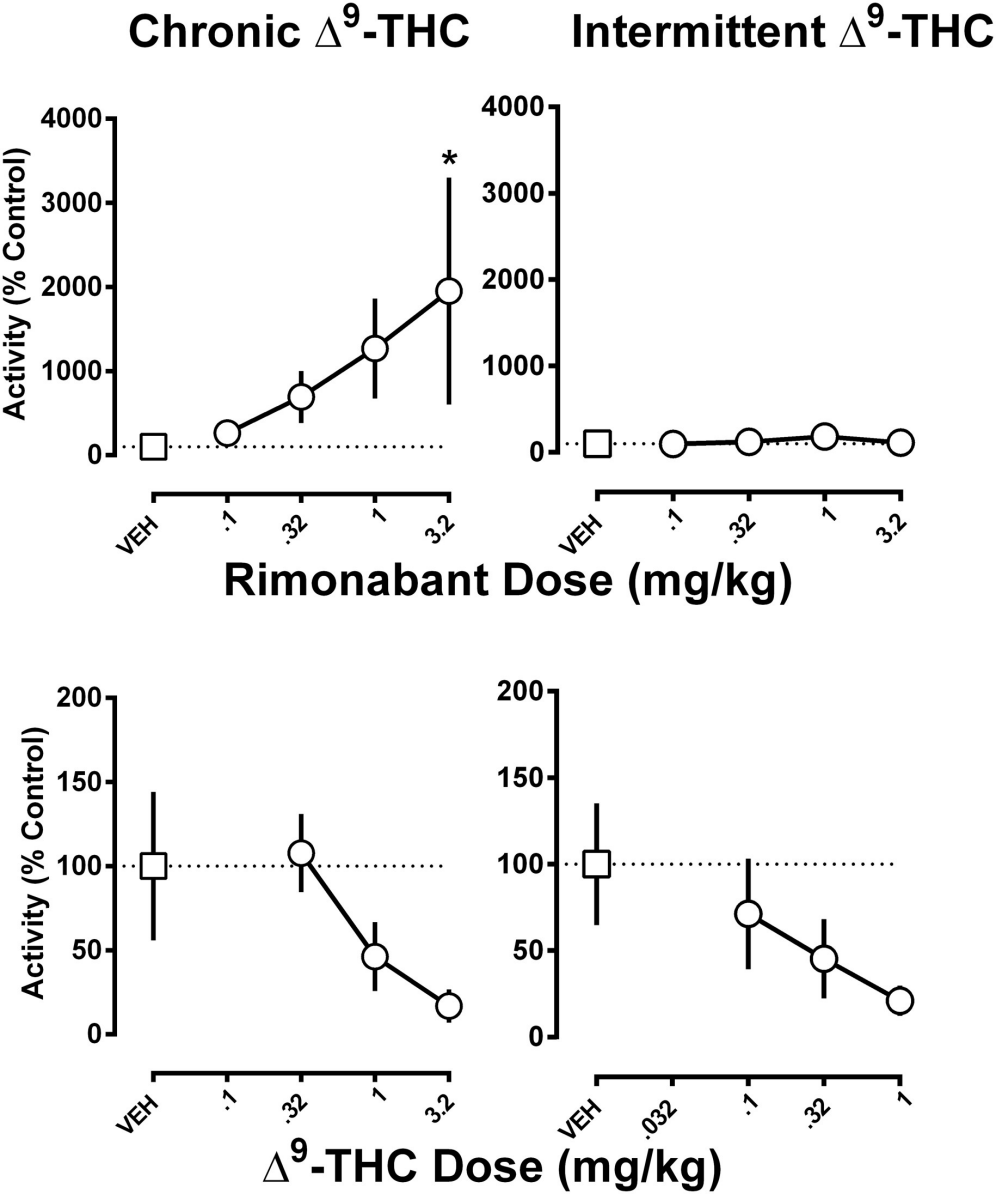
The effects of rimonabant and Δ^9^-THC in chronically and intermittently Δ^9^-THC-treated monkeys. The effects of rimonabant (top panels) and Δ^9^-THC (bottom panels) in monkeys receiving either chronic Δ^9^-THC (left panels) or intermittent Δ^9^-THC (right panels). Activity counts are expressed as a percentage of the control calculated from the average of two separate vehicle tests. The abscissae denote dose in milligram per kilogram of body weight. *P < 0.05 and vs. vehicle. Data reflect mean ± SEM, n = 5 monkeys/group.

When expressed as a percentage of the vehicle control, intravenous rimonabant (0.1–3.2 mg/kg) dose-dependently increased activity in the chronic Δ^9^-THC group [(main effect of treatment: F_2,36_ = 4.17; p<0.05), (Fig 3 top left)], but not the intermittent Δ^9^-THC group [(P = 0.6), (Fig 3 top right)] The dose of 3.2 mg/kg rimonabant significantly increased activity in the chronic Δ^9^-THC group when compared to vehicle (P < 0.05). However, the variance was large due to activity being increased 70-fold in one monkey and unaltered in another monkey. A large amount variance also accounted for no significant main effect of the single dose response curves of Δ^9^-THC, in both the chronic treatment group [(P = 0.2), (Fig 3 bottom left)] and the intermittent treatment group [(P = 0.5), (Fig 3 bottom right)]. The slopes of the Δ^9^-THC dose-response functions in the two groups did not significantly differ from each other. The intercepts also did not significantly differ. However, in this analysis, there was a tendency toward statistical significance (P = 0.1) showing a difference in potency between the two groups to the effects of Δ^9^-THC.

## Discussion

Here we show a novel report that in rhesus monkeys with at least a 2 year history of daily Δ^9^-THC (2 mg/kg), abrupt discontinuation of treatment (i.e., replacing Δ^9^-THC with vehicle) resulted in an immediate (within 24 h) increase in activity. This increase in activity was observed for at least 5 days after Δ^9^-THC discontinuation. Further, here we demonstrate that increased activity in chronically Δ^9^-THC-treated monkeys occurred in a rimonabant dose-dependent manner. Interestingly, intermittent Δ^9^-THC-treated monkeys did not display significantly increased activity due to rimonabant.

In the current study, rimonabant produced a dose-dependent, marked increase in activity but only in monkeys receiving daily Δ^9^-THC treatment. The magnitude of increase was striking, i.e., 20-fold greater than the vehicle baseline. Further, as evidenced by the complete failure of rimonabant to increase activity in a separate group of monkeys that had received only intermittent Δ^9^-THC, the observed increased activity was not related to the direct effects of rimonabant. The marked increase in sensitivity to the effects of a cannabinoid antagonist in animals treated daily with Δ^9^-THC is consistent with a precipitated withdrawal syndrome. Sensitivity to the effects of rimonabant to decrease schedule-controlled responding was similarly increased in rhesus monkeys receiving the same chronic Δ^9^-THC treatment regimen [13], as well as in squirrel monkeys receiving chronic treatment with the cannabinoid agonist AM-411 [16]. The qualitatively similar changes in activity observed not only after abrupt discontinuation of chronic Δ^9^-THC, but also after rimonabant during Δ^9^-THC treatment, further suggest that withdrawal occurred under both conditions. However, the magnitude of change after rimonabant was markedly greater even though activity was cumulated over 2 h, as compared with 24 h in the discontinuation study. These data are consistent with the capacity of an antagonist to produce a greater magnitude of withdrawal as compared with abrupt discontinuation of treatment.

One interpretation is that, instead of withdrawal, the increased activity shown here reflects a return to baseline from decreased activity induced by chronic Δ^9^-THC treatment. However, the systematic decrease in hyperactivity back to baseline levels, which was measured during Δ^9^-THC treatment by day 20 of discontinuation, argues against this interpretation. Further, this time-limited increase in activity is consistent with a withdrawal syndrome, and the current time course is similar to that described for Δ^9^-THC withdrawal in humans [17].

Difficulty sleeping is a reliable component of cannabis withdrawal in humans [18], However, the pattern of activity in the monkeys studied here, expressed as 1-h bins across the 24-h period, shows that increases in activity during discontinuation occurred primarily during the light portion, i.e. 0700–1700 h, of the light-dark cycle. Thus, any sleep disruption that might have occurred due to Δ^9^-THC withdrawal was not evidenced by changes in activity during the dark cycle.

Δ^9^-THC typically decreases activity in rodents [9]. It is likely that tolerance to the activity-decreasing effects of Δ^9^-THC developed in the rhesus monkeys studied while undergoing daily Δ^9^-THC treatment, both before and after the discontinuation period. Intravenous Δ^9^-THC dose-dependently decreased activity in monkeys receiving chronic or intermittent Δ^9^-THC. While there was a tendency for Δ^9^-THC to be less potent in monkeys receiving chronic Δ^9^-THC versus intermittent Δ^9^-THC (Fig 3, compare circle above 0.32 mg/kg in bottom panels), the difference was not large enough to achieve statistical significance given the error variance and sample size. Nonetheless, the current results add to a substantial literature demonstrating tolerance to Δ^9^-THC [19,20]. Further, the current results show that changes in activity in monkeys may be sensitive to both Δ^9^-THC tolerance as well as dependence and are consistent with the observation that withdrawal signs are often opposite to the direct effects of the dependence-inducing drug.

The study described in this body of work employs several distinct advantages for the study of the effects of dependence-inducing drugs. In contrast to rodent studies, non-human primates are unique in that their biology is closely related to that of humans [21]. Here we used an automated approach to measure activity, which holds several advantages over observable behavior scored by an experimenter. Specifically, continuous monitoring of subjects over multiple 24 h periods would be virtually impossible for a single experimenter to conduct. Thus, multiple experimenters would be needed to collect these data, which would entail a laborious process, with the need for correlation analysis of inter-experimenter variability. Further, automated measurement of activity diminishes the likelihood of experimenter bias influencing the data collected. Here we also examine unconditioned behavior, via home-cage activity, with a non-invasive device. One important caveat to many schedule-controlled experiments is that the behavioral task is measured in a non-home cage environment and is reliant on learned behavior. Thus, changes in schedule-controlled behavior can be easily disrupted by drugs which impair learned memory processes.

One caveat in the translation of both non-human primate and rodent studies to humans is that only humans experience the feeling of expectation. Therefore, this feeling cannot contribute to the changes in behavior and physiology comprising a drug withdrawal syndrome as observed in animals. The clinical data describing symptoms and observable signs due to cannabis withdrawal in individuals who abruptly discontinue chronic cannabis use have relied heavily on self-reports. While providing highly useful information on the characteristics of cannabinoid withdrawal, self-reports outside of the clinical laboratory cannot readily differentiate subtle changes in mood, perhaps influenced by expectation, which can result from abrupt discontinuation of any habitual activity. This may account for some of the changes in mood that occur from the physical dependence to cannabis which remains difficult to be independently verified by systematic changes in behavior or physiology. Further, inpatient, double blind, placebo-controlled laboratory studies (e.g. [22,23]) can help to eliminate subject and experimenter bias, which may also hamper the objective quantification the behavioral and physiological effects of cannabis withdrawal.

In summary, these data provide strong evidence for physical dependence to Δ^9^-THC in non-human primates, as evidenced by withdrawal upon abrupt discontinuation of Δ^9^-THC treatment and administration of rimonabant during Δ^9^-THC treatment. The current results are consistent with the results of a previous study demonstrating a disruption of schedule-controlled responding in rhesus monkeys upon abrupt discontinuation of continuous intravenous infusion of Δ^9^-THC for at least 10 days [8]. In that study, the disruption of schedule-controlled behavior was also time-limited, with a time course that was similar to the current study (i.e., 1–2 weeks in duration). Thus, Δ^9^-THC withdrawal is evidenced not only by a change in unlearned behavior as shown in the current study, but also learned behavior [8], suggesting that cannabinoid withdrawal can broadly disrupt behavior. While the exact clinical significance of cannabis withdrawal syndrome remains unclear, the current data demonstrate that physical dependence to Δ^9^-THC, and likely cannabis, clearly develops and should be recognized as a component of cannabis use disorders.

## Abbreviations

**Δ^9^-THC**: Δ^9^-tetrahydrocannabinol
ANOVA: analysis of variance
